# Design, Formulation, and Evaluation of *Aloe vera* Gel-Based Capsaicin Transemulgel for Osteoarthritis

**DOI:** 10.3390/pharmaceutics14091812

**Published:** 2022-08-29

**Authors:** Narayana Charyulu Rompicherla, Punam Joshi, Amitha Shetty, Kalvatala Sudhakar, Hawraz Ibrahim M. Amin, Yachana Mishra, Vijay Mishra, Aqel Albutti, Naif Alhumeed

**Affiliations:** 1 Department of Pharmaceutics, NGSM Institute of Pharmaceutical Sciences, Nitte (Deemed to Be University), Mangaluru 575018, Karnataka, India; 2 School of Pharmaceutical Sciences, Lovely Professional University, Phagwara 144411, Punjab, India; 3 Department of Chemistry, College of Science, Salahaddin University-Erbil, Erbil 44001, Iraq; 4 Department of Medical Biochemical Analysis, Cihan University-Erbil, Erbil 44001, Iraq; 5 Department of Zoology, School of Bioengineering and Biosciences, Lovely Professional University, Phagwara 144411, Punjab, India; 6 Department of Medical Biotechnology, College of Applied Medical Sciences, Qassim University, Buraydah 51452, Saudi Arabia; 7 Deputyship for Research and Innovation, Ministry of Education, Riyadh 11153, Saudi Arabia

**Keywords:** emulsion, capsaicin, arthritis, topical, anti-inflammatory agent, drug delivery

## Abstract

Topical treatments are a potential therapeutic option for the therapy of osteoarthritis, with significant data supporting the effectiveness and safety of topical formulation. Topical gel formulations may offer an alternative to oral formulations to relieve osteoarthritis (OA) pain while decreasing systemic exposure. Topical capsaicin transemulgel may represent an effective and safe alternative. The transemulgel was prepared from aqueous *Aloe vera* gel and Carbopol 934 with capsaicin in clove oil emulsion. The optimized transemulgel of capsaicin showed a pH of 6.1 ± 0.1 and viscosity of 15263–998 cps. Data from in vitro diffusion demonstrated improved permeability properties. The formulation caused no skin irritation when applied topically. The optimal transemulgel spreadability was found to be 20.23 g·cm/s. In vitro and ex vivo studies of the optimized formulation were performed. The skin irritant test was performed on rat skin with an optimized and marketed formulation. Both showed no irritation on the skin. The transemulgel of the capsaicin with *Aloe vera* gel was proven to be effective for osteoarthritis therapy.

## 1. Introduction

Osteoarthritis is the most common degenerative joint disease, affecting more than 20% of the population over 18 years old. Pathological changes in osteoarthritis joints include destruction of articular cartilage, progressive loss, subchondral bone thickening, osteophytes formation, ligament degeneration, menisci of the knee, and hypertrophy of the joint capsule [1]. Treatment includes oral and topical pharmacological agents, patient education, alternative surgery, medicine, physical therapy, and modalities [2]. Topical drug delivery has advantages over conventional routes; in particular, it avoids first-pass metabolism, and is a non-invasive mode of drug delivery with a sustained and controlled release profile [3,4].

Gels as topical drug delivery systems contain a more significant amount of hydroalcoholic liquid in a complex network of solid particles [5]. The main disadvantage of gels is that they are unsuccessful in delivering hydrophobic drugs. To overcome this drawback, transemulgel has been developed to successfully deliver hydrophobic drugs. Transemulgel comprises either a water-in-oil or oil-in-water emulsion mixed with a gelling agent [6]. The advantages of transemulgels are that they provide easy incorporation of hydrophobic drugs into the gel using an oil-in-water emulsion system, which increases stability and controlled release, and has better loading capacity [7,8,9].

Capsaicin, a highly selective and potent agonist for the Transient Receptor potential cation channel subfamily V member 1, is extracted from the dried ripe fruit of *Capsaicin annum* of the family *Solanaceae*. Capsaicin acts as an anti-inflammatory and analgesic agent to treat various diseases such as diabetic neuropathy, rheumatoid arthritis, post-therapeutic neuralgia, and osteoarthritis [10]. Capsaicin is unsuitable for oral administration due to its high first-pass metabolism and gastric irritation. Hence, the topical route is a preferable route [11]. *Aloe vera* (*Aloe barbadensis miller*), a perennial plant belonging to the family *Liliaceae*, is used due to its anti-inflammatory, anticancer, antimicrobial, immunomodulatory, and burn healing effects. It has been used in different commercial products [12,13]. Due to its cooling effect, the burning sensation of capsaicin can be reduced by rubbing the skin with *Aloe vera* gel as a base [14]. Therefore, the present research work aimed to develop and evaluate capsaicin transemulgel using *Aloe vera* gel as a base for the treatment of osteoarthritis.

## 2. Materials and Methods

### 2.1. Materials

Capsaicin was obtained from Plant Lipids Private Limited, Telangana, India. Mineral oil, clove oil, Carbopol 934, Tween 80, Span 80, triethanolamine, propylene glycol, methylparaben, propylparaben, and liquid paraffin were purchased from Loba Chemie, Mumbai. All the other chemicals were procured from HiMedia Lab, Mumbai, India. All the solvents were of High-Performance Liquid Chromatography (HPLC) grade.

### 2.2. Drug and Polymer Interaction Studies

Under Fourier Transform Infrared (FTIR) spectroscopy, five scans were run for clove oil, Tween 80, triethanolamine, capsaicin, and Carbopol 934 to check the compatibility between drug and polymer at a scanning range of 400–4000 cm^−1^ with a speed of 2 mm s^−1^ at a resolution of 4 cm^−1^ at room temperature. The wavenumber of a characteristic peak of the physical mixture was compared with the pure sample and interpreted [15].

### 2.3. Solubility Studies

An excess of capsaicin (3000 µg/mL) was added individually to 10 mL of oils (clove oil and mineral oil), surfactants (Tween 80 and Span 80), and co-surfactants (triethanolamine and propylene glycol) in a 100 mL volumetric flask. Then, the mixture was kept in a shaking water bath at 37 ± 0.5 °C for 48 h at 100 rpm. The sample was then centrifuged at 3000 rpm for 15 min. After centrifugation, 1 mL of the supernatant fluid was withdrawn from each mixture. The absorbance was determined using UV-Visible spectrophotometrically after 10 mL dilution with phosphate buffer saline (PBS) pH 6.8 at λ_max_ 281 nm [16].

### 2.4. Phase Study

Pseudo-ternary phase diagrams were constructed to determine the emulsions’ existing region. Based on capsaicin solubility studies, oil, surfactant, and co-surfactant were selected to create a phase diagram. A mixture of surfactant and co-surfactant (S_mix_) in various weight ratios (1:1, 2:1, and 3:1) was added to each group and sonicated (Sonics Sonicator, Newtown, CT, USA) for 5 min with a frequency of 20 kHz at 25 ± 1 °C. S_mix_ and oil were thoroughly mixed in different weight ratios ranging from 9:1 to 1:9 (i.e., 9:1, 8:2, 7:3, 6:4, 5:5, 4:6, 3:7, 2:8, and 1:9) for each phase diagram. Pseudo-ternary phase diagrams were constructed using slow aqueous phase titration [17,18,19].

### 2.5. Preparation of Capsaicin Loaded Transemulgel

The preparation of transemulgel included three steps. In the first step, the oil phase of the emulsion was prepared by dissolving capsaicin (12.5 mg) in the mixture of clove oil, Tween 80, and triethanolamine. Then, water phase was slowly added to the oil phase while stirring continuously with a magnetic stirrer at 2000 rpm for 10 min. In the second step, the gel base was prepared by dispersing Carbopol 934 in 15 mL of distilled water by stirring on the magnetic stirrer. Methylparaben (25 mg) was dispersed in 5 mL of distilled water and heated in a water bath to dissolve properly (first solution). Then, the solution was cooled, and polyethylene glycol (PEG) 4000 (2 g) was added to the first solution by proper mixing. *Aloe vera* juice (5 mL) was added to this mixture. Finally, all mixed ingredients were added to the Carbopol 934 in an appropriate amount with constant stirring to obtain the gel. Finally, the third step included the incorporation of o/w emulsion into the gel base in the ratio of 1:1 to get a uniform transemulgel [8,20,21,22].

### 2.6. Optimization by Design of Experiment

The effect of various process factors on the preparation of transemulgel was investigated using the Design of Experiment (DoE) approach. The independent factors selected were the ratio of S_mix_, oil, and percentage of Carbopol, and the dependent variables included drug content studies (%) and in vitro diffusion studies (%). Based on the screening study, it was noted that all the other ingredients and parameters had no observable influence on the properties of the formed transemulgels and remained constant in further study. The experimental conditions were optimized as per the runs generated by the Design Expert^®^ Software (Version 11.0.3.0; Statease, Minneapolis, MN 55413, USA) using Central Composite Design (CCD). The factors selected and the levels for the experiment are given in Table 1. A total of 17 runs (trial batches) were generated for transemulgel (Table 2) [23,24].

### 2.7. Determination of Drug Content in Transemulgel Formulations

Drug content estimation was carried out by dissolving 1 g of transemulgel in 100 mL of freshly prepared PBS (pH 6.8) and analyzed spectrophotometrically by a UV-Visible Spectrophotometer at 281 nm [25,26].

### 2.8. In Vitro Diffusion Studies

**Figure 1:**
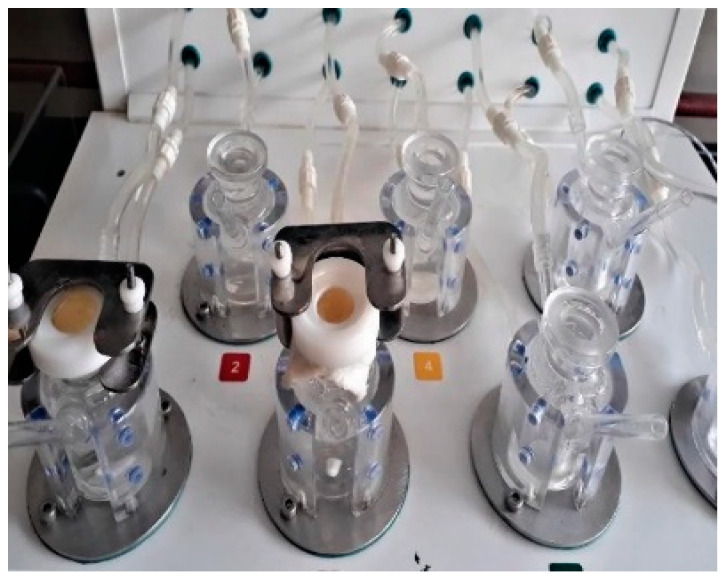
Modified vertical Franz diffusion cell apparatus.

The in vitro drug release was performed using a modified vertical Franz diffusion cell apparatus (Figure 1) [27,28,29].

An accurately weighed quantity of 1 g of transemulgel was placed on the cellophane membrane (donor compartment) and the whole setup was partially immersed in the dissolution medium, PBS. The dissolution medium was mixed at 50 rpm at a temperature of 37 ± 0.5 °C using a magnetic stirrer. At pre-determined time intervals, i.e., 15, 30, 60, 90, 120, 150, 180, 210, 240, 270, 300, 330, 360, 390, 420, 450, and 480 min, 1 mL of the sample was withdrawn to measure the absorbance at 281 nm using a UV-Visible Spectrophotometer. The sink condition was maintained by replacing the withdrawn sample with the same volume of fresh PBS [27,28,29].

### 2.9. Optimization and Evaluation of Optimized Transemulgel Formulation

A numerical optimization technique using the desirability function approach was used to obtain an optimum formulation. To achieve the desired responses of the formulation, the appropriate levels of constraints (target) were chosen.

#### 2.9.1. Physical Appearance

The developed optimized transemulgel formulation was visually inspected for its color, appearance, and consistency [30,31].

#### 2.9.2. Determination of pH

A digital pen pH meter was used to determine the pH of the transemulgel. The pH was determined by dispersing 2.5 g of transemulgel formulation in 25 mL of distilled water [32].

#### 2.9.3. Viscosity Measurement

The viscosity of the transemulgel was measured using a Brookfield viscometer fitted with spindle no. S 63 at 50 rpm for 10 min [33].

#### 2.9.4. Spreadability

**Figure 2:**
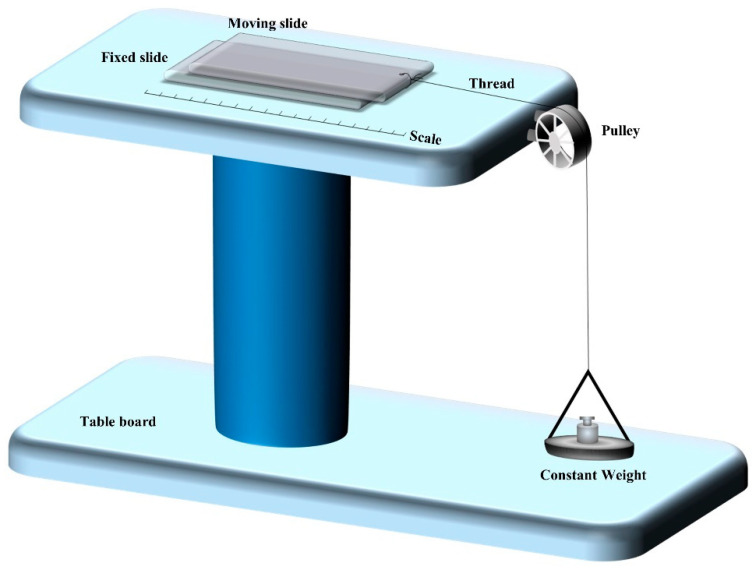
Spreadability test using the wooden block method.

A modified apparatus consisting of a wooden block was used to conduct the spreadability test. This consisted of a wooden block attached to a pulley at one end. The spreading was measured based on ‘Slip’ and ‘Drag’ characteristics of transemulgel. A ground glass slide was fixed on the wooden block (Figure 2).

Briefly, the transemulgel (2.5 g) was placed between two transparent glass slides. A weight of 100 g was placed on the top of the two slides for 5 min to expel air and to provide a uniform film of the transemulgel between the two slides. A measured quantity of weight (80 g) was placed in the pan attached to the pulley with the help of a hook, and the upper slide was expected to fall off. The time taken for the above slide to move the distance of 7.5 cm and detach from the lower slide under the weight direction was noted [34,35,36]. Spreadability was calculated by using the following formula:

(1) $$\mathrm{Spreadability}\;(S)=\left(m\times l\right)⁄t$$

where S = spreadability, m = weight tied to the upper slide, l = length of the glass slide, t = time taken in seconds for the complete detachment of slides from each other.

#### 2.9.5. Drug Content Studies

Drug content studies were carried out by dissolving 1 g of transemulgel in a 100 mL volumetric flask and making the volume up to 100 mL with freshly prepared PBS (pH 6.8). Solution (1 mL) was withdrawn and diluted up to 10 mL with PBS (pH 6.8). The drug content in transemulgel was estimated spectrophotometrically using a UV-Visible Spectrophotometer at 281 nm [37,38]. Experiments were performed in triplicate (n = 3) to estimate the drug content uniformity.

### 2.10. Ex Vivo Diffusion Studies

An ex vivo skin permeation study was carried out using a fresh porcine skin membrane. Briefly, the porcine skin membrane was placed on the donor compartment with the help of a clamp and partially dipped in dissolution medium, PBS pH 6.8 filled in the receptor compartment. An accurately weighed quantity of transemulgel (1 g) was placed on the porcine skin membrane of the donor compartment. The dissolution medium (PBS pH 6.8) was mixed using a magnetic stirrer at 50 rpm. The temperature was maintained at 37 ± 0.5 °C. A sample (2 mL) was withdrawn at a pre-determined time interval and analyzed using a UV-Visible Spectrophotometer at 281 nm. Fresh PBS pH 6.8 (2 mL) was added to replenish the withdrawn sample to maintain the sink condition [39,40,41,42,43,44].

### 2.11. Drug Release Kinetic Studies

An in vitro release kinetic study was utilized to examine the mechanism of drug release. The data of drug release can be fitted using the following equations [45]:

(2) $$\text{Zero-order equation}:Q=Q_{0}+{\;k}_{0}t$$

where Q_0_ = initial amount of drug, Q = cumulative amount of drug release at time “t”, (released occurs rapidly after drug dissolves), K_0_ = zero order release constant, t = time in hours

(3) $$\mathrm{First}\;\mathrm{order}\;\mathrm{equation}:\;\mathrm{logC}={\mathrm{logC}}_{0}-\mathrm{kt}/2.303$$

where C_0_ = initial amount of drug, C = cumulative amount of drug release at time “t”, K = first order release constant, t = time in hours.

(4) Higuchi model equation: Q=kHt12

where Q = cumulative amount of drug release at time “t”, k_H_ = Higuchi constant, t = time in hours

(5) Korsmeyer–Peppas model equation: MtM=kmtn

where M_t_ = amount of drug released at time ‘t’, M = total amount of drug in dosage form, k_m_ = kinetic constant, n = diffusion or release exponent, t = time in hours

### 2.12. Comparison of Experimental Results with Predicted Responses of Optimized Formulation

Observed values after performing the test were then compared with the predicted values obtained from the software, and the relative % error was determined.

### 2.13. Stability Studies

Stability studies were performed to determine the stability of capsaicin in transemulgel. The optimized formulation was stored in a collapsible tube at 30 °C/65% relative humidity (RH) and 40 °C/75% RH in the humidity chamber for 28 days. Samples were collected following 15 days and 28 days of storage and assessed for physical appearance, pH, spreadability, and drug content. To prove the stability of the transemulgel, the similarity index was calculated between in vitro diffusion rates.

### 2.14. Skin Irritation Test

The skin irritation studies were carried out on the Wistar rats as per the protocol approved by the Institutional Animal Ethics Committee (IAEC) of NGSM Institute of Pharmaceutical Sciences, Nitte (Deemed to be University), Mangaluru-575018, India (Reg. No. NGSM/IAEC/2020-21/193; dated 25 June 2020) for studies involving animals. Briefly, 12 healthy Wistar rats of either sex weighing 200–240 g were selected and divided into four groups i.e., Group I (optimized formulation), Group II (marketed formulation), Group III (controlled subjects i.e., blank transemulgel), and Group IV (standard subject i.e., plain drug). Twenty-four hours before the application of the formulation, the selected Wistar rats were individually weighed again, their hairs were removed from the dorsal portion of the skin, and methylated spirit was then applied as an antiseptic to the shaved region with the aid of cotton wool to prevent infection caused by bacteria. Optimized transemulgel, marketed formulation, blank transemulgel, and plain drug were applied to the depilated area (0.5 g/6 cm^2^) of animals of respective groups. Animals were observed for skin irritation per the Organization for Economic Co-operation and Development (OECD) guidelines at pre-determined time intervals, i.e., 1 h, 24 h, 48 h, 72 h, and 7 days [46,47,48,49,50,51].

### 2.15. Statistical Analysis

Unless explicitly illustrated, all data are expressed as mean standard deviation (SD). The two-way ANOVA calculated the statistical importance of variations between more than two groups. Values between *p* < 0.05 and *p* < 0.001 were significant and highly significant, respectively.

## 3. Results and Discussions

### 3.1. Drug and Polymer Interaction Studies

**Figure 3:**
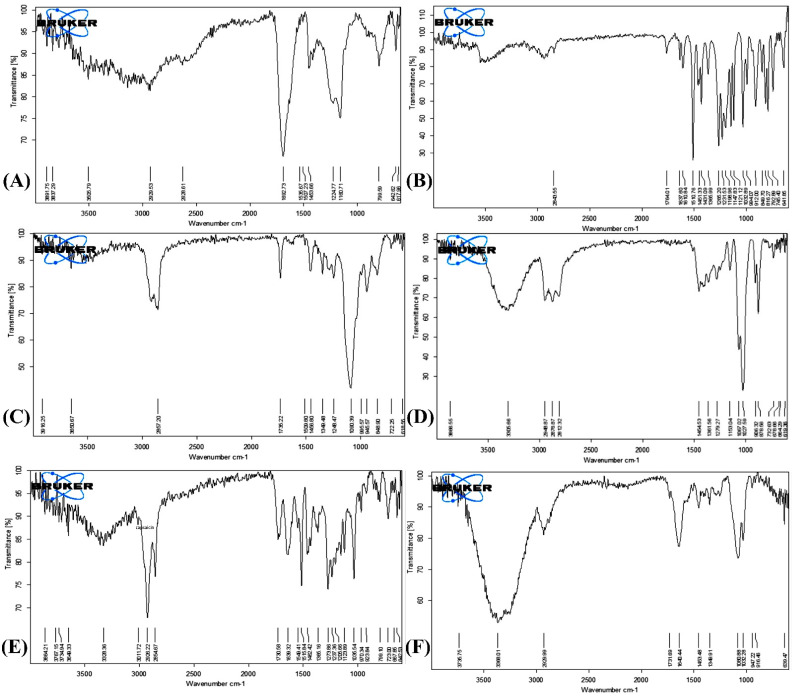
FTIR spectrum of: (**A**) Carbopol 934; (**B**) clove oil; (**C**) Tween 80; (**D**) triethanolamine; (**E**) capsaicin; (**F**) optimized transemulgel formulation.

The absorption peaks of FTIR spectra of different ingredients such as Carbopol 934, clove oil, Tween 80, and triethanolamine are represented in Figure 3A–D, respectively. Figure 3E represents the absorption peak of the pure drug, capsaicin. The optimized formulation (Figure 3F) showed no shift and no disappearance of characteristic peaks, suggesting no interaction between the drug and the other ingredients used.

The FTIR spectrum of capsaicin showed the characteristic peaks: N-H stretch (3326.36 cm^−1^), aliphatic C-H stretch (2926.22 cm^−1^ and 2864.67 cm^−1^), C=O stretch (1639.32 cm^−1^), aromatic C-C stretch. (1549.41 cm^−1^) and out-of-plane C-H bending (799.10 cm^−1^), N-H bending and C-N stretch (Amide II) (1515.16 cm^−1^), asymmetric C-O-C stretch (1273.86 cm^−1^), and C-O stretch (1206.66 cm^−1^). In the formulation spectrum, characteristic peaks were visible, indicating the compatibility with ingredients. Transemulgel formulation spectrum showed absorption bands in the range of 3300–3368.01 cm^−1^ due to characteristic stretching vibrations (N-H) of amino acids. The C-H stretch was visible in transemulgel at 2299.90 cm^−1^, N-H bending, and C-N stretch (Amide II) at 1498.16 cm^−1^. All samples showed a range of 1453.43–1640.44 cm^−1^ due to the presence of (C-C) stretching vibration in the aromatic ring and bending out-of-plane (C-H) at a range of (639.47 cm^−1^) (Table 3).

### 3.2. Solubility Studies

The maximum solubility of capsaicin was found to be 0.630 ± 0.010 mg/mL, 0.609 ± 0.006 mg/mL, and 0.680 ± 0.007 mg/mL in Tween 80, triethanolamine, and clove oil, respectively. Thus, based on the capsaicin solubility, Tween 80 (surfactant), triethanolamine (co-surfactant), and clove oil were selected to prepare the emulsion. All experimental values were derived in triplicate (n = 3) [52].

### 3.3. Phase Study

**Figure 4:**
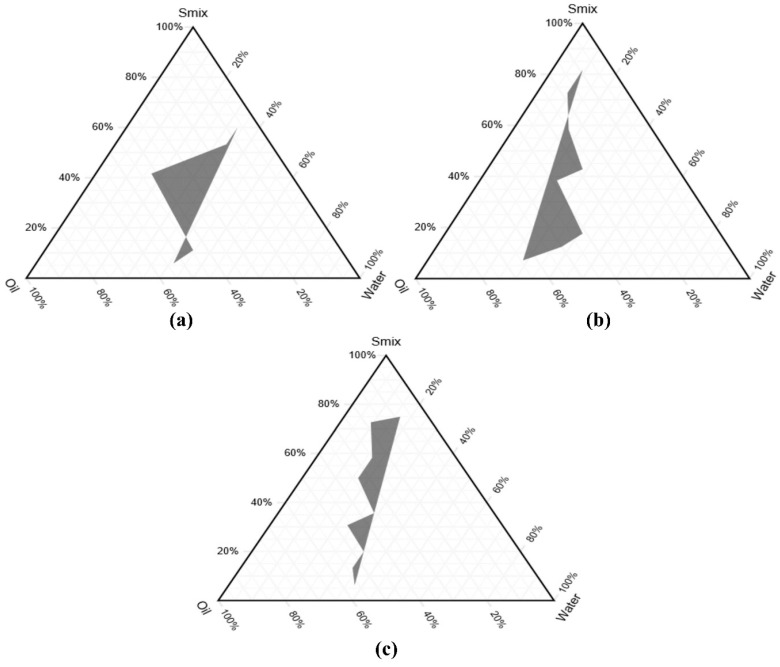
Pseudo-ternary phase diagram of: (**a**) S_mix_ ratio 1:1; (**b**) S_mix_ ratio 2:1; (**c**) S_mix_ ratio 3:1.

The construction of a phase diagram helps to determine the concentration range of components for existing emulsions. As depicted in Figure 4a–c, the black area shows the o/w emulsion region in the different ratios (i.e., 1:1, 2:1, and 3:1) of S_mix_ to oil. The largest emulsion region was obtained for the surfactant: co-surfactant ratio of 1:1, and the smallest emulsion area was obtained for the ratio of 3:1. As the concentration of S_mix_ increases, the quantity of water required to obtain turbidity decreases. Tween 80 and triethanolamine at the ratio of 1:1 resulted in a greater emulsion region. Therefore, this system was chosen for further study.

### 3.4. Optimization by Design of Experiment (DoE) and Evaluation of Prepared Transemulgel Formulations

The transemulgels were optimized using 2^3^ levels of factorial randomized central composite design using Design Expert. Seventeen trial batches for transemulgel were prepared and evaluated for the drug content and in vitro diffusion, and the responses for transemulgel obtained from DoE. The drug content and in vitro drug diffusion were found to be 86.01–97.08% and 48.29–90.37%, respectively. Maximum drug content was obtained from (S_mix_):70:2.5 (Carbopol), and maximum in vitro diffusion was obtained from (S_mix_):45:2.5 (Carbopol) [52].

### 3.5. Effect of Different Factors on the Selected Responses

#### 3.5.1. Effect of Factors on Drug Content Studies

**Figure 5:**
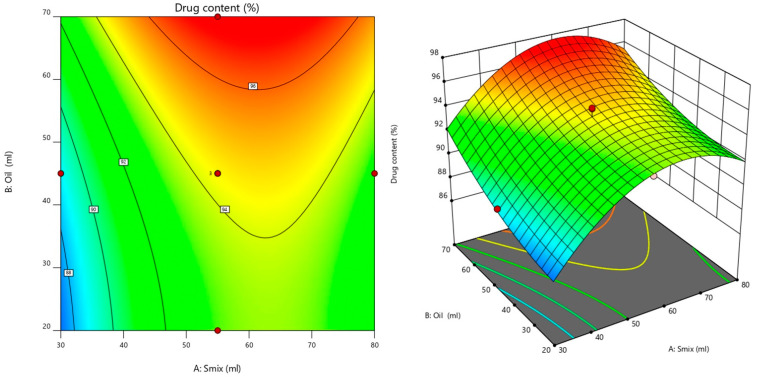
Contour plot and response surface plot for the effect of factors on drug content.

The model developed for the drug content had a *p*-value of 0.0027 and an F-value of 10.33, indicating the model was significant. The value of 0.1605 showed a non-significant lack of fit, implying the model is appropriate to calculate the drug content. Based on the contour plot and response surface plot (Figure 5), it was observed that, as the concentration of S_mix_ and oil ratio increased from −1 to +1, there was a significant increase in the drug content. The S_mix_ and oil ratio significantly affected the drug content of transemulgel, as shown in the following equation.

Drug content = +94.38 + 2.02 A + 2.11 B + 0.4970 C − 0.3837 AB + 0.6338 AC − 0.8787 BC − 3.58 A2 + 0.9177 B2 − 0.0973 C2(6)

where A, B, and C represent the coded values for S_mix_, oil, and Carbopol, respectively.

#### 3.5.2. Effect of Factors on In Vitro Drug Diffusion

The model developed for the diffusion had a *p*-value of 0.0115 and an F-value of 6.40, indicating the model to be significant. The value of 0.0044 showed a non-significant lack of fit, implying the model is appropriate to calculate the diffusion. The amount of S_mix_ and oil ratio significantly affected the diffusion study of transemulgel, as shown in the following equation.

Diffusion = +81.96 + 3.16 A − 1.75 B + 0.6620 C + 1.22 AB + 0.0250 AC − 0.2000 BC − 18.30 A2 − 20.68 B2 + 9.85 C2(7)

where A, B, and C represent the coded values for S_mix_, oil, and Carbopol, respectively.

**Figure 6:**
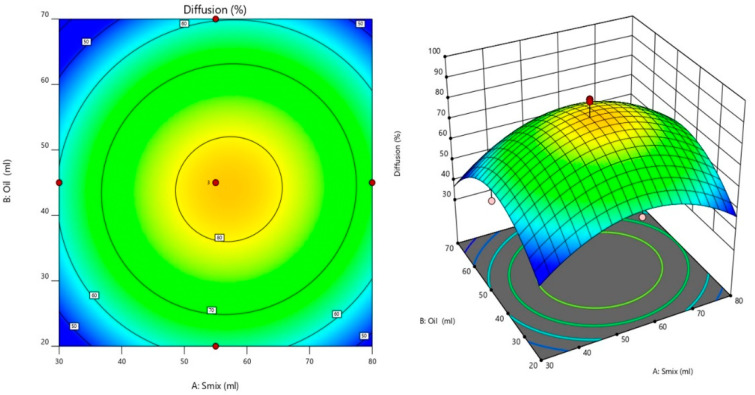
Perturbation plot and response surface plot for the effect of factors on diffusion.

Based on the perturbation plot and response surface plot shown in Figure 6, it can be seen that, as the concentration of S_mix_ and oil ratio increased from −1 to +1, there was a significant decrease in the in vitro diffusion.

### 3.6. Optimization and Evaluation of Optimized Formulation

**Figure 7:**
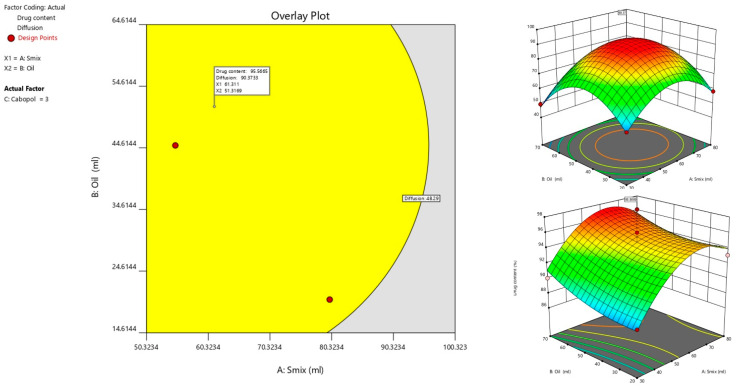
Optimization of the transemulgel formulation.

The criterion for numerical optimization (Table 4) and the optimized transemulgel formulation based on the Design Expert^®^ software is represented in Table 5 and Figure 7.

#### 3.6.1. Physical Appearance

The optimized transemulgel formulation was evaluated for color, appearance, and consistency. The physical appearance of the transemulgel was found to be yellowish with a smooth and glossy appearance. Phase separation was not observed.

#### 3.6.2. Determination of pH

Evaluation of pH is a crucial parameter for topical formulations as it can cause skin irritation if it differs from standard skin pH conditions. The transemulgel pH was found to be 6.1 ± 0.1, closer to the skin pH. Hence, the transemulgel formulation may not cause any irritation when it is externally applied to the skin surface.

#### 3.6.3. Viscosity Measurement

**Figure 8:**
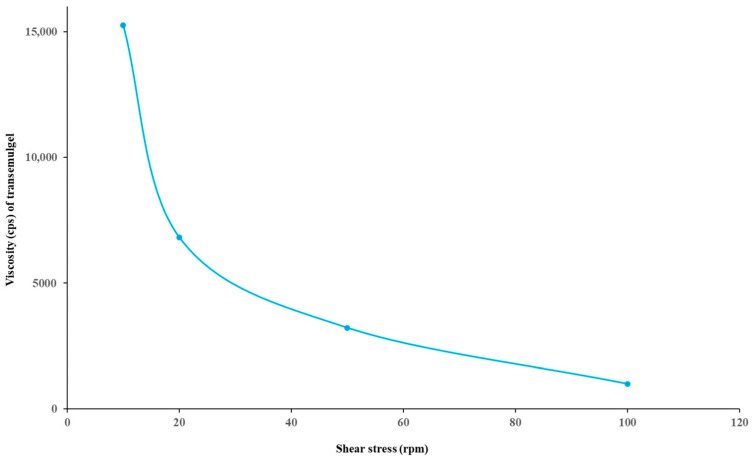
The viscosity of optimized transemulgel (MM10).

Viscosity is a significant rheological parameter that is related to the mechanical and physical properties, such as spreadability, consistency, and hardness of the preparation. In turn, it is associated with the ease of product removal from the container, ease of application on the skin surface, and the feel of the product on the applied area. The viscosity of the optimized formulation was found to be in the range of 15,263–998 cps. The results obtained are given in Figure 8. The obtained results and the pattern of the plot showed that, as the shear rate increased, viscosity decreased, and vice versa.

#### 3.6.4. Spreadability

The spreadability of the gel plays a major role in the application; if spreadability is poor, it eventually hinders the duration of drug residence on the skin, which can contribute to poor bioavailability. The spreadability of optimized transemulgel was found to be 20.23 g·cm/s, which indicates good spreadability.

#### 3.6.5. Drug Content Studies

Uniformity of the drug content is necessary for semi-solid preparation to ensure the homogeneity of the dispersed drug in the entire formulation. The drug content of the optimized transemulgel was found to be 94.5 ± 1.74%. The assessment of drug content also suggested the uniform distribution of the drug. The loss of the drug during formulation processes was insignificant.

#### 3.6.6. In Vitro Diffusion Studies

**Figure 9:**
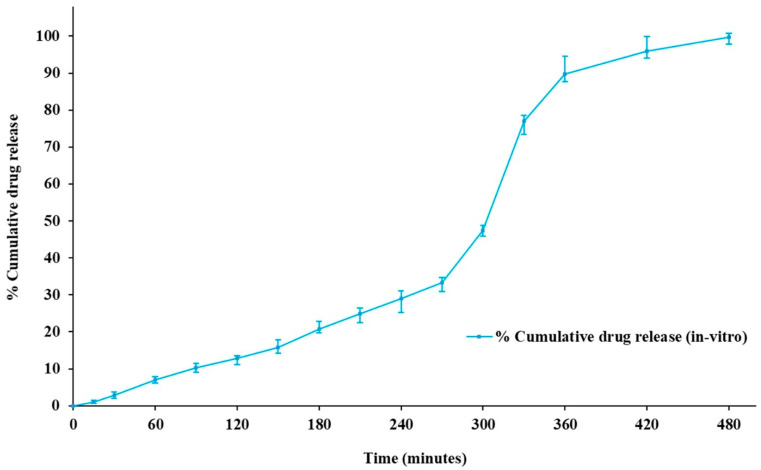
In vitro diffusion studies of optimized transemulgel formulation.

The optimized transemulgel was subjected to in vitro diffusion studies using a cellophane membrane. The cumulative amount of drug release was calculated, and the drug release after 480 min was found to be 99.8 ± 1.23%. Optimized transemulgel of capsaicin followed a sustained drug release pattern (Figure 9).

#### 3.6.7. Ex Vivo Study

**Figure 10:**
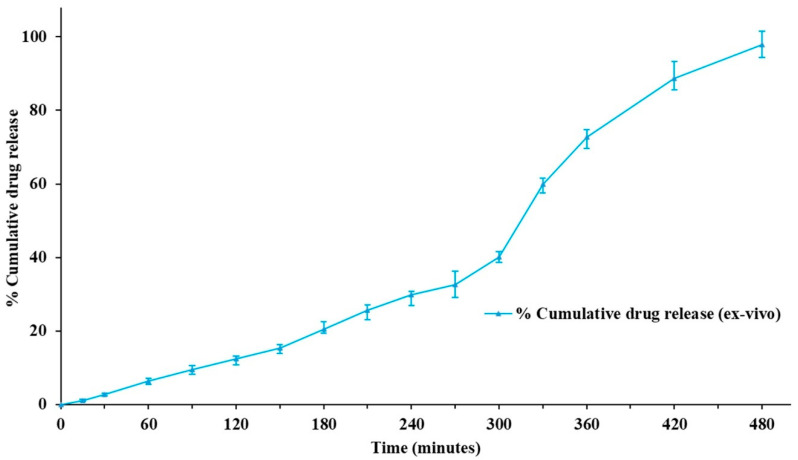
Ex vivo diffusion studies of optimized transemulgel formulation.

The optimized transemulgel was subjected to ex vivo study using a fresh porcine skin membrane. The cumulative drug release was found to be 97.8 ± 3.12% after 480 min. Optimized transemulgel of capsaicin followed a sustained drug release pattern (Figure 10).

#### 3.6.8. Drug Release Kinetics

The various kinetic models were applied to in vitro release data to predict the drug release kinetic mechanism. The release constants were calculated from the slope of appropriate plots, and the regression coefficient (R^2^) was determined. As per data depicted in Table 6, it was found that the zero-order Korsmeyer–Peppas model best explained the in vitro drug release of the optimized transemulgel formulation, and the drug transport mechanism followed super case-II transport.

#### 3.6.9. Stability Study

Data from the stability studies are represented in Table 7 and Table 8. The optimized transemulgel formulation was found to be stable upon storage for 28 days, and no change was observed in their physical appearance, pH, spreadability, and drug content. After storage of 28 days, the formulation was subjected to in vitro diffusion studies. The difference and similarity factors between dissolution profiles of optimized formulation before and after storage were found to be 4 and 82, respectively. The difference factor was found to be less than 15, and the similarity factor was found to be more than 50, indicating similarity between the dissolution profile before and after storage.

#### 3.6.10. Comparison of Experimental Results with Predicted Responses of Optimized Formulation

Table 9 lists the predicted and experimental values of all the response variables and the percentage error. Upon comparison of the observed responses with the predicted responses, the mean percentage error was found to be 0.89. Thus, the low magnitude of error indicated an excellent fit of the model.

### 3.7. Skin Irritation Test

**Figure 11:**
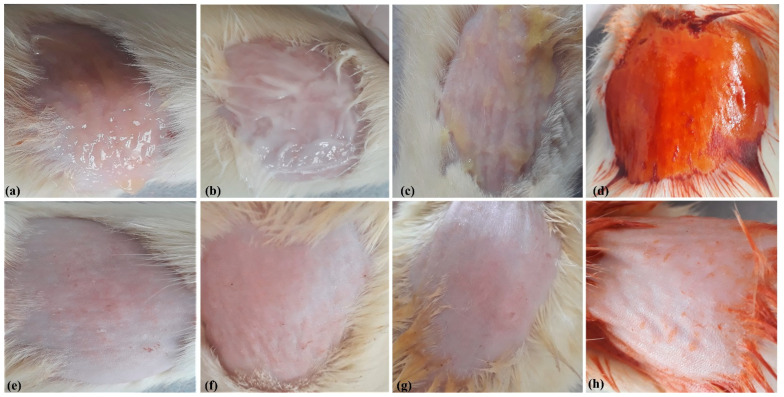
Skin irritation test; (**a**) optimized formulation subject, Group I; (**b**) marketed formulation, Group II; (**c**) controlled subjects (blank transemulgel), Group III; (**d**) standard subject (plain drug), Group IV. After applying the different formulations on skin irritation test animal subjects, their effects are shown in (**e**–**h**), respectively.

Group I, Group II, Group III, and Group IV were used for optimized formulation, marketed formulation, control group (blank transemulgel), and standard group (plain drug), respectively. The optimized formulation was applied to rat skin. After inspecting visually, the prepared transemulgel was compatible with rat skin and showed no irritation. The rats were observed after 0, 24, and 72 h and the readings were noted. There was no sign of erythema or oedema formation in the entire period of 72 h with capsaicin-loaded transemulgel, but Group II showed an irritation in terms of color change in the skin and redness, as shown in Figure 11. As suggested in the results, the side effect of the standard group can be overcome by transemulgel. From the results, it can be concluded that the capsaicin-loaded transemulgel was found to be irritation-free.

## 4. Conclusions

Capsaicin emulsion was successfully formulated and then incorporated into the *Aloe vera* gel as the base to reduce skin irritation caused by capsaicin. Capsaicin is an active component that acts as an anti-inflammatory and analgesic agent. The major drawback in formulating capsaicin for topical drug delivery is that capsaicin is a poorly water-soluble drug. Thus, incorporation of capsaicin into o/w emulsion and then into gel increased the solubility. Based on the results, the transemulgel showed better permeation and no irritation to skin, and maintained the sustained release profile. The incorporation of *Aloe vera* into the emulgel reduces the irritability of capsaicin. The optimized transemulgel of capsaicin was analyzed by composite design based on the parameters of surfactant, oil, and Carbopol gel polymer concentration. The spreadability of the transemulgel was good enough to spread over the skin easily. The transemulgel followed zero order and Korsmeyer–Peppas models with transport mechanism case-II transport. Therefore, overall, the results showed that transemulgel can be a promising formulation for the application of capsaicin to relieve pain in osteoarthritis patients.

## Figures and Tables

**Table 1:** Independent and dependent factors selected and levels for the experiment.

Independent Factors	Name	Level (−1)	Level (0)	Level (+1)
A	S_mix_ ratio (% *v/w*)	30	55	80
B	Oil ratio (% *v/w*)	20	45	70
C	Carbopol (% *w/w*)	2	2.5	3
Dependent factors				
A	Drug content			
B	Diffusion studies			

**Table 2:** 2^3^ Levels of factorial randomized central composite design experimental trial batches for transemulgel.

Formulation Code	Factor 1	Factor 2	Factor 3
	S_mix_ Ratio	Oil Ratio	Carbopol (%)
F1	0	0	+1
F2	0	0	0
F3	−1	+1	+1
F4	+1	−1	−1
F5	+1	−1	+1
F6	0	−1	0
F7	−1	0	0
F8	0	0	0
F9	−1	−1	+1
F10	−1	−1	−1
F11	+1	0	0
F12	0	+1	0
F13	−1	+1	−1
F14	+1	+1	−1
F15	0	0	0
F16	0	0	−1
F17	+1	+1	+1

**Table 3:** Various peaks obtained from FTIR spectra of capsaicin, clove oil, Tween 80, triethanolamine, Carbopol 934, and optimized formulation.

Functional Groups	Reported Groups (cm^−1^)	Compounds					
		Drug (Capsaicin) (cm^−1^)	Clove Oil (cm^−1^)	Tween 80 (cm^−1^)	Triethano- Lamine (cm^−1^)	Polymer (Carbopol 934) (cm^−1^)	Optimized Formulation (cm^−1^)
C-H stretching (Alkane)	2840–3000	2926.22	2843.55	2857.20	2876.87	2929.53	2929.99
C=O stretching (Aldehyde)	1720–1740	1730.58	-	1735.22	-	-	1731.69
O-H bending (Phenol)	1310–1390	1365.16	1365.99	1349.48	1361.56	-	1349.91
S=O stretching (Sulfoxide)	1030–1070	1035.54	1032.89	-	1067.02	-	1032.28
O-H stretching	3200–3700	3328.36	-	3650.67	3305.66	3505.79	3368.01
C=C stretching (Conjugated alkene)	1600–16700	1639.32	1637.60	-	-	-	1640.44

**Table 4:** Criteria for numerical optimization.

Parameters	Goal	Lower Limit	Upper Limit	Lower Weight	Upper Weight	Importance
A: S_mix_ ratio	Is in range	−1	1	1	1	1
B: Oil ratio	Is in range	−1	1	1	1	1
C: Carbopol	Is in range	−1	1	1	1	1
Y1: Drug Content	Target = 95.18	86.01	97.08	1	1	1
Y2: Diffusion	Target = 90.3	48.29	90.37	1	1	1
MM10 (optimized)	Concentration of S_mix_ (%)		Concentration of oil (%)		Carbopol (%)	
	61.31		51.32		3	

**Table 5:** Observed responses for transemulgels obtained from DoE.

Formulation Code	Factor-1	Factor-2	Factor-3	Response-1	Response-2
	S_mix_-Ratio	Oil-Ratio	Carbopol (%)	Drug Content (%)	Diffusion (%)
F1	0	0	+1	96.01	86.15
F2	0	0	0	95.02	90.37
F3	−1	+1	+1	90.03	49.32
F4	+1	−1	−1	91.07	56.41
F5	+1	−1	+1	93.08	58.34
F6	0	−1	0	93.03	60.16
F7	−1	0	0	89.02	55.12
F8	0	0	0	94.01	89.19
F9	−1	−1	+1	88.03	54.17
F10	−1	−1	−1	86.01	52.27
F11	+1	0	0	92.09	60.28
F12	0	+1	0	97.08	50.48
F13	−1	+1	−1	94.07	48.29
F14	+1	+1	−1	95.05	57.26
F15	0	0	0	95.08	90.15
F16	0	0	−1	92.07	85.55
F17	+1	+1	+1	96.09	58.44

**Table 6:** Drug release kinetics.

Optimized transemulgel	Model	R^2^	n
	Zero-order	0.998	-
	First order	0.649	-
	Higuchi	0.732	-
	Korsmeyer–Peppas	0.991	1.175

**Table 7:** Stability studies of optimized transemulgel formulation.

Time Period	Physical Appearance		pH		Spreadability (g·cm/s)		Drug Content (%)	
	30 °C/ 65% RH	40 °C/ 75% RH	30 °C/ 65% RH	40 °C/ 75% RH	30 °C/ 65% RH	40 °C/ 75% RH	30 °C/ 65% RH	40 °C/ 75% RH
Before storage	No phase separation	-	6.1 ± 0.1	-	20.23	-	94.5%	-
After 15 days	No phase separation	No phase separation	6.1 ± 0.31	6.1 ± 0.41	20.20	20.18	94.5%	94.0%
After 28 days	No phase separation	No phase separation	6.1 ± 0.18	6.1 ± 0.76	20.13	19.01	94.3%	94.0%

**Table 8:** Difference and similarity factors of dissolution profile of optimized formulation.

Time (min)	Before Storage	After 28 Days		Difference Factor	Similarity Factor
		30 °C/65% RH	40 °C/75% RH		
360	89.68 ± 1.38	89.18 ± 1.35	87.89 ± 1.18	4	82

**Table 9:** Selected solution and % error between the predicted and the observed values.

Factors			Responses	
A	B	C	Drug Content (%)	Diffusion (%)
			**Predicted**	
61.31	51.32	3	95.56	90.37
			**Observed**	
			94.37	89.68
Relative % error			1.19	0.59

## Data Availability

Data are contained within the article.
